# Role of GM-CSF in lung balance and disease

**DOI:** 10.3389/fimmu.2023.1158859

**Published:** 2023-04-04

**Authors:** Yingzi Chen, Fan Li, Mengqing Hua, Meng Liang, Chuanwang Song

**Affiliations:** ^1^ Department of Immunology, School of Laboratory Medicine, Bengbu Medical College, Anhui, China; ^2^ Anhui Province Key Laboratory of Immunology in Chronic Diseases, Bengbu Medical College, Anhui, China; ^3^ Department of Biotechnology, School of Life Science, Bengbu Medical College, Anhui, China

**Keywords:** GM-CSF, alveolar macrophages, surfactant, lung homeostasis, lung disease

## Abstract

Granulocyte-macrophage colony-stimulating factor (GM-CSF) is a hematopoietic growth factor originally identified as a stimulus that induces the differentiation of bone marrow progenitor cells into granulocytes and macrophages. GM-CSF is now considered to be a multi-origin and pleiotropic cytokine. GM-CSF receptor signals activate JAK2 and induce nuclear signals through the JAK-STAT, MAPK, PI3K, and other pathways. In addition to promoting the metabolism of pulmonary surfactant and the maturation and differentiation of alveolar macrophages, GM-CSF plays a key role in interstitial lung disease, allergic lung disease, alcoholic lung disease, and pulmonary bacterial, fungal, and viral infections. This article reviews the latest knowledge on the relationship between GM-CSF and lung balance and lung disease, and indicates that there is much more to GM-CSF than its name suggests.

## Introduction

Granulocyte-macrophage colony-stimulating factor (GM-CSF, or CSF2), a member of the CSF family of hematopoietic growth factors, was originally identified as a stimulus that induces the differentiation of bone marrow precursor cells into granulocytes and macrophages (1). Studies now show that GM-CSF has a wide range of biological activities in innate and adaptive immunity, and it plays a key role in many autoimmune and inflammatory diseases (2, 3). Given that the most prominent phenotypic features of GM-CSF-deficient mice are surfactant alveolar accumulation and impaired alveolar macrophages (AMs) function (4), this review mainly focuses on the role of GM-CSF in lung balance and lung disease.

## Biology and receptor of GM-CSF

GM-CSF is a polypeptide growth factor with a molecular weight of 23 kDa, and its encoding gene is located at 5q22-31, with four exons and 2.5 kb in length. Human GM-CSF contains 127 amino acid residues and is present in serum and most tissues (5). GM-CSF-producing cells can be myeloid cells, such as monocytes/macrophages, eosinophils, neutrophils. They can also be non-myeloid cells, such as epithelial cells, endothelial cells, chondrocytes, fibroblasts, and even tumor cells (6–10). A variety of stimuli can induce GM-CSF production; in T cells, IL-1β, IL-12, IL-23, and prostaglandin E2 can stimulate the production of GM-CSF (11–14). The combination of IL-1 and TNF-α can induce GM-CSF production in a variety of cells, such as endothelial cells, smooth muscle cells, chondrocytes, and fibroblasts, T and B cells (15). In lymphocytes, GM-CSF production requires activation of the transcription factor NF-AT (16, 17), and the production of GM-CSF by adipocytes requires activation of NF-κB (18), indicating that a variety of transcription factors can mediate GM-CSF production. Certain cytokines such as IFN-γ, IL-4, and IL-10, and drugs such as cyclosporine A and glucocorticoids can inhibit GM-CSF production (19–23). The target cells of GM-CSF include a variety of myeloid cells, such as monocytes, macrophages, eosinophils, neutrophils, and dendritic cells (DCs) (3). GM-CSF has various effects on the biological activity of myeloid cells, such as cell activation and proliferation, increasing their chemotaxis and adhesion, promoting the production of pro-inflammatory factors, and improving cell phagocytosis and antigen presentation functions (24–29). Tissues of GM-CSF-deficient mice, especially the lungs, have increased susceptibility to pathogenic microorganisms (30–33). This indicates that GM-CSF plays an important role in maintaining immune function.

**Figure 1:**
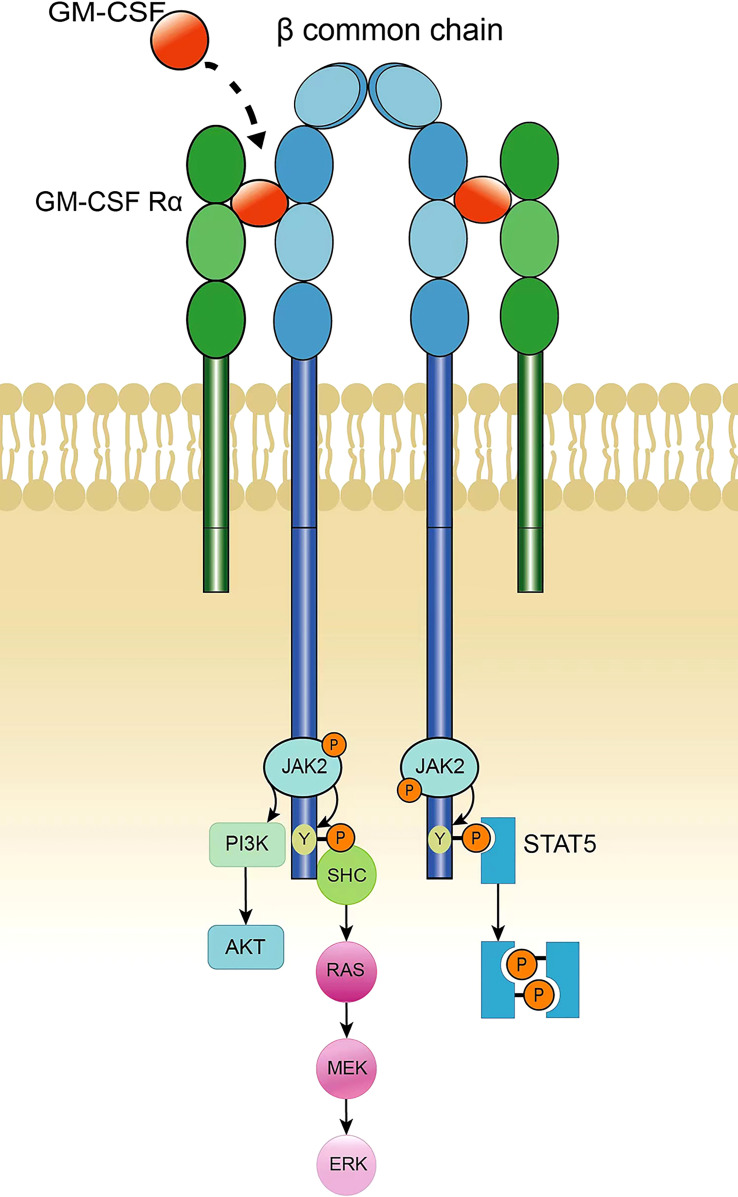
GM-CSF receptor and signaling.

GM-CSF receptors consist of heterodimers, including α chains that specifically bind GM-CSF and β chains responsible for signal transduction; β chains are common chains of IL-3 and IL-5 receptors. Neither the α nor β chains of the GM-CSF receptor contain a tyrosine kinase catalytic domain, although the β chain is linked to the tyrosine kinase JAK2 (34, 35). After the GM-CSF receptor α chain binds to GM-CSF, it then binds to the β chain to form a multimer. The polymerization of the receptor leads to the activation of JAK2, and activated JAK2 phosphorylates the tyrosine residues on the β chain. Phosphorylated tyrosine recruits STAT-5 containing the SH2 domain, and JAK2 activates STAT-5, thereby activating the JAK-STAT pathway. In addition, JAK2 can cause the activation of PI3K, initiating the PI3K-Akt pathway. Phosphorylated tyrosine on the β chain of the GM-CSF receptor recruits the adaptor protein SHC to activate RAS and initiate the MAPK signaling pathway to induce nuclear signaling (**Figure 1**). The JAK2-STAT-5 pathway mainly controls cell differentiation and inflammatory signaling, whereas PI3K signaling promotes cell proliferation and survival, and the MAPK pathway is involved in cell growth, proliferation, and differentiation (6, 36, 37).

The GM-CSF receptor consists of a dimer of α and β common chains. Binding of GM-CSF to the α chain results in polymerization of the β subunit of the receptor; this causes the transactivation of JAK2, which phosphorylates multiple tyrosine sites on the β chain. Phosphorylated tyrosine recruits STAT5, which is then phosphorylated by JAK2. The two phosphorylated STAT5 molecules form a homologous dimer that translocates into the nucleus and initiates target gene transcription. In addition, activation of PI3K can be accomplished by JAK2-mediated phosphorylation of the regulatory subunit p85, thereby activating the PI3K-Akt pathway. Activation of the MAPK pathway is triggered by the recruitment of SHC by a phosphorylated tyrosine on the β subunit, which catalyzes RAS activation, leading to continuous activation of RAF, MEK, and ERK. JAK2-STAT5 signaling controls differentiation and inflammation, PI3K promotes cell proliferation and survival, and MAPK is involved in cell growth, proliferation and differentiation.

## GM-CSF and alveolar macrophages

AMs are lung-specific tissue-resident macrophages that play an important role in maintaining alveolar homeostasis by engulfing inhaled bacteria and removing excess surfactant and cellular debris (38, 39). AMs mainly originate from fetal liver (FL) monocytes, but yolk sac (YS) monocytes and circulating monocytes can also be used as AMs precursors, indicating that there is a compensatory mechanism for the development of AMs (40). When FL monocytes differentiate into AMs in response to inflammation, the body can compensate by inducing YS monocytes or circulating monocytes to differentiate into AMs. Studies analyzing GM-CSF-deficient and GM-CSF receptor β chain-deficient mice in the 90s demonstrated a functional link between GM-CSF and AMs (4, 41). Lung histology of GM-CSF-deficient mice show amorphous eosinophilic accumulation and foamy AMs, similar to the pathological features of human pulmonary alveolar proteinosis (PAP). However, exogenous GM-CSF infusion can restore the AMs population in GM-CSF-deficient mice, suggesting that GM-CSFs play a key role in AMs development (42). GM-CSF is involved in the development of AMs from the embryonic stage, and the level of GM-CSF in the lungs is higher after the 17.5th day of the embryo. Increased levels of GM-CSF in the lungs are temporally consistent with the differentiation of AMs precursors into AMs (40). GM-CSF induces the expression and functional activity of PPAR-γ in fetal monocytes, and PPAR-γ regulates the developmental process of AMs as a master transcription factor. The amount of AMs in PPAR-γ deficient mice is relatively low and they show abnormalities that are similar to the abnormalities of AMs in GM-CSF-deficient mice and GM-CSF receptor β chain-deficient mice. This suggests that the GM-CSF signaling-induced PPAR-γ plays an important role in the development of AMs (43). Shibata et al. showed that the transcription factor PU.1 not only mediated the dependence of the final differentiation of AMs on GM-CSF, but also regulated innate immune functions such as pathogen killing of AMs and the catabolism of surfactants (44).

Normally, the sources of lung GM-CSF include both immune and non-immune cells, such as type 2 innate lymphocytes (ILC2), basophils, and epithelial cells (9, 45, 46). Among neonatal immune cells, ILC2 produces the highest level of GM-CSF, followed by γδT cells (45). The depletion of lymphocytes or basophils has no effect on the amount of AMs in neonatal and adult lungs, suggesting that GM-CSF derived from immune cells is dispensable for the development and maintenance of AMs. When GM-CSF in alveolar type 2 epithelial cells (AT2s) is depleted, AMs in neonatal and adult mice are almost completely depleted, indicating an integral role of AT2-derived GM-CSF in AMs development (47).

## GM-CSF and surfactant homeostasis

Pulmonary surfactant is composed of approximately 90% lipids and 10% proteins. Approximately 80–90% of the lipids are phospholipids, and surfactant-related proteins include SP-A, SP-B, SP-C, and SP-D. These proteins are involved in the intracellular transport of phospholipid components and contribute to the maintenance of surfactant properties in the alveoli (48, 49). Surfactant phospholipids and proteins are synthesized and secreted by AT2s (50, 51). Surfactants form single and multiple layers at the gas-liquid interface to reduce surface tension and prevent alveolar collapse. Surfactants become inactive small aggregate particles through mechanical or biological action, and are absorbed, reused, or decomposed by AT2s and AMs (52).

Studies on the relationship between GM-CSF and surfactant balance have demonstrated the significant accumulation of phospholipids and proteins in the lung of GM-CSF- deficient mice (53–55). Lung histology show a large number of inclusion bodies and enlarged AMs with amorphous eosinophilic substances. These characteristics are similar to the typical manifestations of human PAP (56, 57). GM-CSF is required for cholesterol clearance and that reduced cholesterol clearance is a primary macrophage defect in PAP pathogenesis (58). The expression of GM-CSF in the lungs completely corrects the alveolar protein deposition caused by endogenous GM-CSF gene-targeted ablation, and likewise, inhaled rather than systemic administration of GM-CSF corrects PAP in GM-CSF-deficient mice (57, 59–62). These findings suggest that the presence of GM-CSF locally in the lungs is necessary and sufficient to restore homeostasis of pulmonary surfactant.

**Figure 2:**
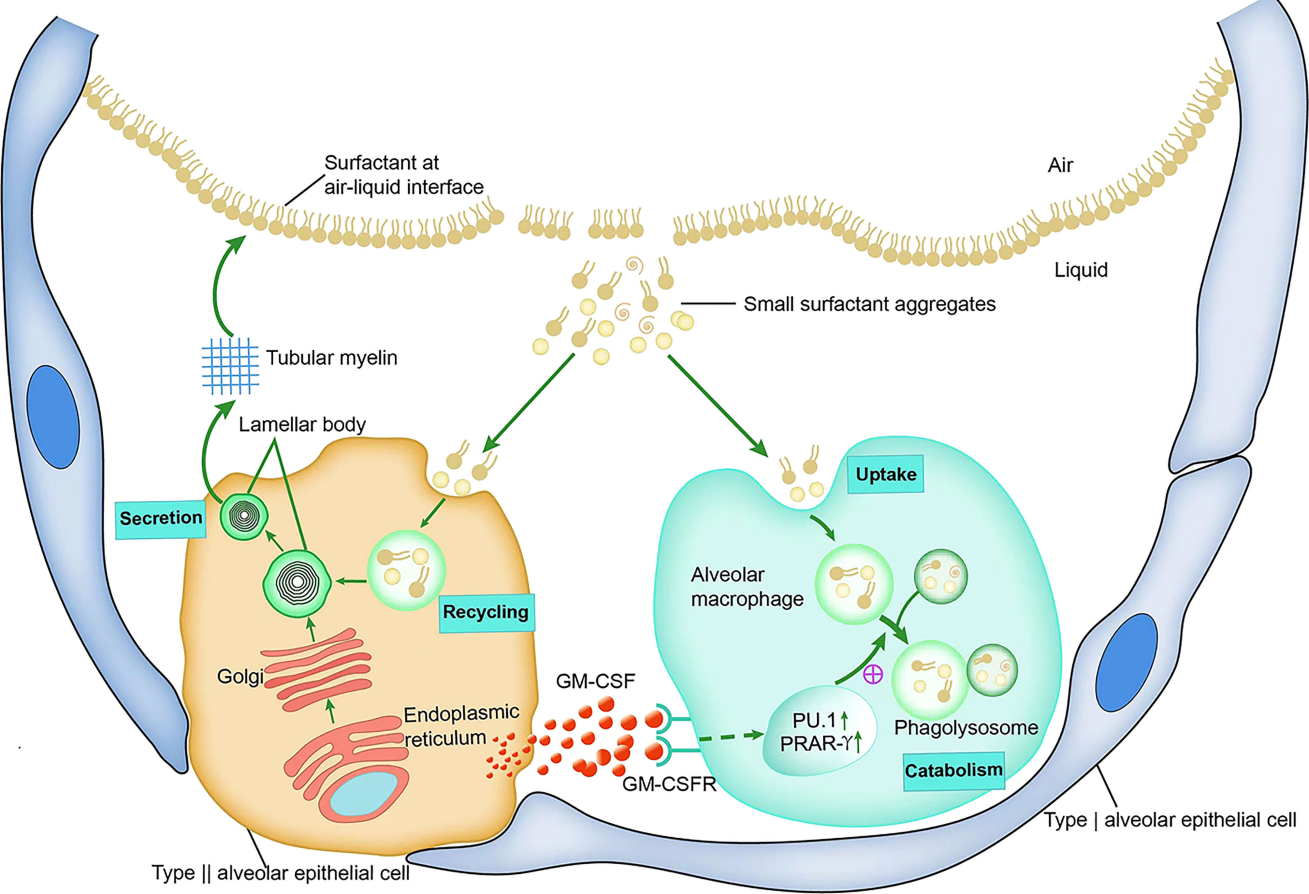
GM-CSF and surfactant homeostasis.

Metabolic studies in GM-CSF-deficient mice have shown that the accumulation of surfactant in the alveoli of GM-CSF-deficient mice is due to the fact that the lack of GM-CSF signaling impairs the catabolism of the surfactant, whereas it does not directly alter its phospholipid and protein synthesis or secretion (5). *In vitro* studies of AMs isolated from GM-CSF-deficient mice identified significant defects in the catabolism of SP-A and surfactant phospholipids, as well as a marked reduction in the degradation of SP-A and DPPC by AMs (63). In addition, mice with increased GM-CSF expression in the lungs show an increased surfactant catabolic rate in AMs, whereas the uptake of surfactant components by GM-CSF-deficient mouse AMs is not hindered (5). These results suggest that in the absence of GM-CSF signaling, the main defect in surfactant homeostasis is caused by insufficient catabolism of surfactant proteins and lipids by AMs (5), which may be related to the need for GM-CSF signaling from AT2 for the development and differentiation of AMs. This also shows that there is a mutually beneficial symbiotic relationship between AT2 and AMs. AT2-derived GM-CSF guides the development of AMs by promoting PU.1 and PPAR-γ expression; in turn, mature AMs are essential for breaking down the surfactants produced by AT2 and maintaining the balance of the alveolar environment (**Figure 2**).

Surfactant proteins and phospholipids are synthesized in AT2s, transported through the endoplasmic reticulum to the Golgi apparatus for processing, and then transported to the lamellar body. The lamellar bodies are secreted into the alveolar fluid, where they form tubular myelin. Tubular myelin forms a surface-active phospholipid film at the air-liquid interface that spreads along the surface of the alveoli. Small aggregates of surfactants that have been inactivated by mechanical and biological processes are partially absorbed and reused by alveolar AT2s, and the other part is taken up and broken down by AMs. GM-CSF, which mainly originates from AT2s in the alveoli, promotes the differentiation and function of AMs by binding to the AMs surface GM-CSF receptor and initiates intracellular signals to stimulate the expression and activation of the transcription factors PU.1 and PPAR-r, thereby enhancing the catabolism of small aggregates.

## GM-CSF and interstitial lung disease

Interstitial lung disease (ILD) includes a group of heterogeneous lung diseases characterized by pulmonary parenchymal inflammation and fibrosis. GM-CSF is involved in the progression of pulmonary fibrosis. GM-CSF production in bronchoalveolar lavage fluid is increased in patients with pulmonary fibrosis (64). GM-CSF stimulates macrophages to produce profibrotic cytokines and can directly induce airway smooth muscle cell fibrosis (3, 65). Autoimmune or inflammatory mechanisms play an important central role in the pathogenesis of connective tissue disease-associated with ILD (CTD-ILD) (66, 67).

SKG mice, which are a model of autoimmune arthritis, treated with yeast polysaccharides develop chronic progressive ILD. These mice exhibit massive lung infiltration of Th17 cells, GM-CSF-producing CD4+ T cells, and CD11b+Gr1+ neutrophils, accompanied by pulmonary fibrosis. Naive SKG mouse T cells differentiate into GM-CSF-producing cells; these enhance macrophage production of IL-6 and IL-1β, thereby promoting the differentiation of IL-17A and/or GM-CSF-producing T cells and the infiltration of neutrophils into the lungs. Neutralization of GM-CSF blocks the development of ILD, whereas neutralization of IL-17A does not, suggesting that GM-CSF, rather than IL-17A, is critical for the development of ILD in SKG mice (68). Kwon et al. also showed that GM-CSF played an important role in ILD development, but these authors believed that IL-17A+GM-CSF+ neutrophils were the main inflammatory cells infiltrated in the lungs of curdlan-treated SKG mice (69).

## GM-CSF in allergic disease

GM-CSF mediates allergen-induced Th2 sensitization and airway eosinophil inflammatory responses (70). The use of GM-CSF receptor α (Csf2ra)-deficient mice illustrates that GM-CSF signaling, although not necessary for the development of eosinophils in normal mice, promotes eosinophil accumulation in the lungs and aggravates airway inflammation in the allergic asthma model; this may be associated with GM-CSF-induced chemotaxis and could promote eosinophil survival (71). In a mouse model of asthma, allergen-stimulated airway epithelial cells release GM-CSF, which activates DCs and prolongs eosinophil survival (72). Intranasal administration of GM-CSF-neutralizing antibodies during allergen inhalation significantly reduces airway hyperreactivity and inhibits airway inflammation (73). In a chronic allergic airway inflammation model, although the GM-CSF signal could not regulate neutrophil migration, GM-CSF promoted antigen uptake by lung DCs, processed and transported to draining lymph nodes, thus enhancing the Th2/Th17 immune response, which in turn increased the recruitment of granulocytes to the lung and aggravated lung inflammation (74).

## GM-CSF signaling and alcoholic lung

AMs in alcoholics are defective in cell adhesion, cytokine production, and phagocytosis (75, 76). Alcohol may affect lung immune function by affecting GM-CSF signaling (77, 78). Although the GM-CSF concentration in the blood and lungs of alcoholics is not markedly different from that in non-alcoholics, chronic alcohol exposure significantly decreases the levels of the GM-CSF receptor for AMs (77), and this decrease is mediated by a decrease in the activity of the transcription factor PU.1 (44, 78). GM-CSF increases PU.1 activity in alcohol-exposed AMs and restores agonist-induced cytokine production and phagocytosis in AMs (78).

The mechanism underlying the decreased activity of PU.1 in alcoholic lungs may be associated with a decrease in antioxidant defense mechanisms (79). Chronic alcohol exposure decreases the lung levels of Nrf2, a major transcription factor that regulates the expression of many antioxidant genes in cells. Nrf2 regulates the expression of PU.1 in the lungs, suggesting that the decrease in antioxidant response function may be related to the decrease in GM-CSF activity in alcoholic lungs and lung immunodeficiency (76).

Chronic alcohol exposure can also lead to reduced levels of alveolar epithelial GM-CSF receptors, suggesting that alcohol can extensively disrupt GM-CSF signaling in the lungs (78), and that the absence of GM-CSF signaling disrupts the epithelial barrier function in the distal lung epithelium. This could explain why alcoholics are more likely to develop acute respiratory distress syndrome (ARDS) based on increased alveolar wall permeability (76, 80, 81). Therefore, the lack of GM-CSF signaling plays a key role in pulmonary dysfunction in patients with alcoholism, and GM-CSF replacement therapy has been used to improve lung complications in patients with alcoholism (82).

## GM-CSF in lung infections

In bacterial, fungal, and viral infections of the lungs, first-line defense cells such as AMs and airway epithelial cells (AECs) initiate an immune response to the microbial challenge (83). Because prolonged or excessive immune responses can damage the respiratory tract, it is important to modulate the inflammatory response to maintain an appropriate airway immune response (84, 85).

## GM-CSF and Streptococcus pneumoniae infection

*Streptococcus pneumoniae* is the most common pathogen causing pneumonia and fatal pneumonia (86). Recombinant GM-CSF delivered through the airway improved the defense response against Group B streptococcus in GM-CSF-deficient mice, and wild-type mice cleared Group B streptococcus faster when receiving aerosol GM-CSF (32). Pulmonary delivery of GM-CSF 2 to 4 weeks prior to infection significantly reduced mortality in *S. pneumoniae* infected mice, and this increased survival was accompanied by an increase in the expression of inducible nitric oxide synthase and antibacterial activity in lung sentinel cells, as well as a significant decrease in caspase-3-dependent apoptosis and secondary necrosis. This suggests that prophylactic delivery of GM-CSF into the lungs triggers a lasting immunostimulatory response that improves alveolar immunity against pneumococcus (87). In mice intranasally infected with influenza A virus (IAV), followed by treatment with atomized recombinant GM-CSF and re-infection with *S. pneumoniae*, inhalation of GM-CSF had significant survival benefits against the secondary attack of *S. pneumoniae* and significantly reduced the incidence of *S. pneumoniae* bacteremia (88).

## GM-CSF and pulmonary fungal infections

Patients with acquired GM-CSF deficiency are susceptible to Cryptococcus and other opportunistic fungi (89–91). In GM-CSF-deficient mice, control of cryptococcal lung infection is impaired. Deficiency of GM-CSF decreases the following processes: 1) total lung leukocyte recruitment; 2) Th2 and Th17 responses; 3) the number of CD11b (+) DCs and macrophages; and 4) the activation and localization of DCs and macrophages in alveoli. These results indicate that GM-CSF promotes the local activation, differentiation, accumulation, and alveolar localization of DCs and macrophages in the lungs of cryptococcus lung-infected mice, suggesting that GM-CSF is a core factor in the protective immune response against mycosis (91).

## GM-CSF and influenza virus

Influenza virus (IV) infection is a common cause of acute respiratory failure in the pediatric intensive care unit (92). The primary target cells for human IV invasion are AECs (93). GM-CSF secreted by AECs plays an important role in preventing influenza-induced pneumonia (94). The ability of GM-CSF-deficient mouse AMs to clear pathogenic microorganisms is damaged, which reduces resistance to influenza virus infection. However, alveolar GM-CSF increases the proliferation and resistance of mouse AMs, thus protecting mice from the deadly IV infection (85). Pulmonary CD103^+^DC is a key factor mediating GM-CSF-dependent lung protection after IV infection (95, 96). After IV infection, lung CD103^+^DC is activated and expanded, and GM-CSF mediates its migration and antigen presentation in draining mediastinal lymph nodes. This is associated with better viral clearance and Ag-specific T-cell recruitment. Therefore, GM-CSF-dependent crosstalk between IV-infected AECs and CD103^+^DCs is important for effective IV clearance (96).

## GM-CSF and SARS-COV-2

Coronavirus disease 2019 (COVID-2), which is caused by severe acute respiratory syndrome coronavirus type 2 (SARS-CoV-2), has evolved into a global pandemic and treatments are urgently needed (97–100). GM-CSF, an important hematopoietic growth factor and pro-inflammatory cytokine, has attracted attention as a therapeutic target in COVID-19 (99). The circulating levels of GM-CSF are increased in patients with COVID-19 compared with healthy controls (101, 102). Patients with COVID-19 have an increased percentage of white blood cells expressing GM-CSF (103). As an important immunomodulatory cytokine, GM-CSF helps clear respiratory microorganisms by activating AMs, suggesting that it could help clear SARS-CoV-2 early in the course of COVID-19 (104, 105). However, GM-CSF is harmful as part of the cytokine storm in the late stage of severe lung injury caused by COVID-19. Therefore, blocking GM-CSF or the GM-CSF receptor may be an effective treatment strategy to block the progression of acute respiratory failure in patients with COVID-19 by reducing the cytokine storm and the infiltration of inflammatory myeloid cells (102).

## Discussion

GM-CSF is a cytokine of multiple cellular origins and pleiotropy, and in the lungs, it is mainly derived from AT2s (47). TNF-α and IL-1β can also induce the production of GM-CSF by endothelial cells and fibroblasts in the lung. The GM-CSF produced by these cells in response to stimulation helps guide leukocyte infiltration into the tissues (106–109). Quiescent alveolar epithelial cells produce very little GM-CSF, whereas AT2s are the main source of GM-CSF in the alveoli in response to stimulation by factors such as infection or inflammation (47). However, the molecular mechanism of GM-CSF production remains unclear. GM-CSF can affect the plasticity of AT2s in an autocrine manner and influence the differentiation of AT2s to alveolar type 1 epithelial cells (AT1s) by regulating cell cycle genes (110–112). GM-CSF also decreases the susceptibility of AT2s to oxidative stress damage, protecting AT2s from the effects of hyperoxia (112).

GM-CSF induces the transcription factors PU.1 and PPAR-γ to drive AM differentiation and maintenance (43, 44). Moreover, GM-CSF can drive the innate immune function of AMs such as pathogen clearance (44). GM-CSF-deficient mice are prone to respiratory infections, and restoring GM-CSF expression reverses this susceptibility, suggesting that GM-CSF-driven AMs play an important role in the innate immunity of the lungs.

Pulmonary surfactant is synthesized and secreted by AT2s. GM-CSF deficiency has little effect on the production or secretion of surfactant lipids or proteins from AT2s, although it has a significant inhibitory effect on the ability of AMs to clear surfactant substances (proteins and lipids) (104, 113–115). The decrease in surfactant clearance of AMs is largely due to disruption of signaling events mediated by the transcription factor PU.1 or PPAR-γ (116, 117). Therefore, GM-CSF signaling drives the differentiation and development of AMs through the PU.1 and PPAR-γ transcription factors and mediates the catabolism of surfactant proteins and lipids. It should be noted that AT2s can also be involved in the catabolism of surfactants. Therefore, future studies will confirm whether GM-CSF affects the catabolism of surfactant in AT2s.

In addition to maintaining surfactant balance and promoting AM differentiation and metabolism, GM-CSF signaling plays an important role in lung infection or inflammation. GM-CSF protects against pneumococcal pneumonia in the lungs by upregulating iNOS expression through PU.1 and STAT5 signals, which contribute to the antibacterial activity of AMs against pneumococcal pneumonia (87). In GM-CSF-deficient mice, control of fungal lung infections is impaired (118). GM-CSF is involved in host defense against fungal lung infections by promoting the differentiation, accumulation, activation and alveolar localization of lung DCs and macrophages (91). Lung CD103^+^DC is a key factor mediating GM-CSF-dependent lung protection after IV infection. IV induces the production of GM-CSF in alveolar epithelial cells, which leads to the activation and migration of CD103^+^DCs to drainage mediastinal lymph node (MLN). This increases the accumulation of IFN-γ+CD4+ and IFN-γ+CD8+T cells to the alveoli, accelerates IV clearance, and mediates recovery from epithelial damage (96). In the early stages of SARS-CoV-2 infection, the role of GM-CSF may be protected as it helps limit virus-related damage. Therefore, the inhaled formulation of human recombinant GM-CSF, sargramostim, is being tested in patients with acute hypoxic respiratory failure associated with COVID-19. In the later stages of SARS-CoV-2 infection, the severity of the disease appears to be driven by improper release of cytokines such as GM-CSF. These inflammatory mediators are involved in inflammatory lung injury, making patients prone to respiratory failure and eventually leading to ARDS. Therefore, inhibition of GM-CSF signaling may be a reasonable treatment in the late stages of COVID-19 (102).

GM-CSF was first known as a hematopoietic growth factor that is used to boost bone marrow production; however, as described in this review, its function goes far beyond what was identified in earlier studies. In addition to promoting the metabolism of pulmonary surfactant and the maturation and differentiation of AMs, GM-CSF plays a key role in lung bacterial, fungal, and viral infections, interstitial lung disease, allergic lung disease, alcoholic lung, and other disease states. Therefore, GM-CSF would be an attractive target for future research on lung balance and lung disease.

## Author contributions

YC and FL: writing original draft, searching literature. MH: searching literature, reviewing and editing. ML and CS: supervision, writing, reviewing, and editing. All authors discussed the article and approved the submitted version.
